# Supplementary figures and images for: Improving accuracy of GPT-3/4 results on biomedical data using a retrieval-augmented language model
Source: PLOS Digit Health. 2024 Aug 21;3(8):e0000568. doi: 10.1371/journal.pdig.0000568 (PMC11338460; doi:10.1371/journal.pdig.0000568)

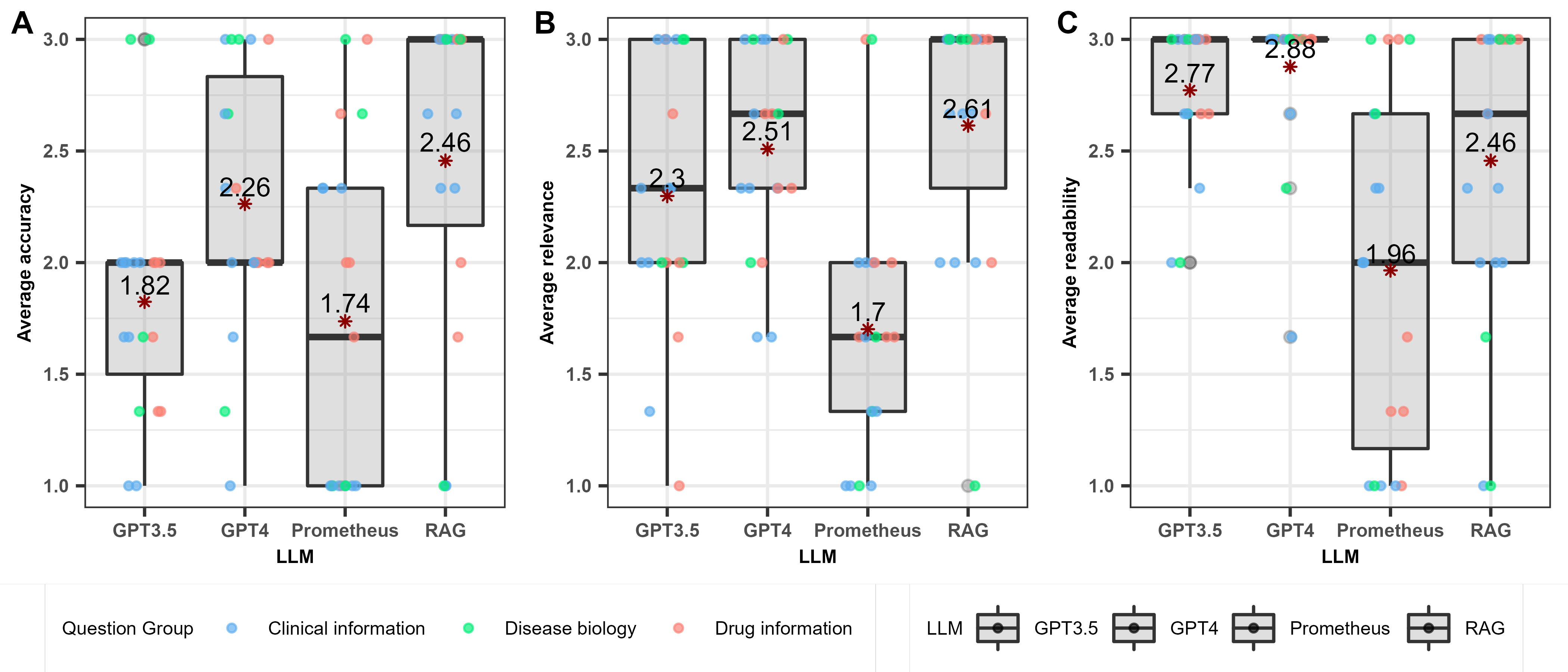

Supplement: S1 Fig — Boxplot of average score per question for each LLM model. Each point represents the average (A) accuracy, (B) relevance, and (C) readability score for a single question (out of 19 total). Points are colored by the question category. (TIFF) [file pdig.0000568.s001.tiff]
