# Supplementary material for: Improving accuracy of GPT-3/4 results on biomedical data using a retrieval-augmented language model
Source: PLOS Digit Health. 2024 Aug 21;3(8):e0000568. doi: 10.1371/journal.pdig.0000568 (PMC11338460; doi:10.1371/journal.pdig.0000568)
Supplement: Attachment — Submitted filename: 2024 PDIG-D-23-00346 Soong et al - Response to Reviewers.docx [file pdig.0000568.s003.docx]

Dear Dr. Sarmiento and PLOS Digital Health reviewers,

Thank you for taking the time to review our manuscript, “Improving accuracy of GPT-3/4 results on biomedical data using a retrieval-augmented language model”. We’ve revised our manuscript based on the suggested edits proposed by both reviewers. We’ve highlighted below what we did to address each reviewer comment, and where in the manuscript we’ve addressed these comments with new additions or modifications (Line numbers are in reference to the “2024 PDIG-D-23-00346 Soong et al - Manuscript.docx” file). We look forward to your feedback on this revised manuscript, and hope that with these changes the manuscript now meets PLOS Digital Health’s publication criteria.

Best regards,

Sriram Sridhar

## Reviewer #1:

In this paper, the authors aim to reduce to practice the use of LLMs as a knowledge agent in the pharmaceutical/biotech setting by evaluating various LLMs, including a retrieval augmented one, in their ability to answer several canonical questions about DLBCL. Expert reviewers scored the LLM outputs in terms of accuracy, relevance, and readability and find that the retrieval augmented model performs best on aggregated, although uniformly. This work represents an early case study in the utility of LLMs in the biotech/pharmaceutical setting and so its significance is highly time dependent, meaning with each passing quarter year models will improve, domain-specific models will evolve, and key challenges will be addressed, e.g. alignment and model updating.

The findings reported herein are useful for practioners and the main novelty lies in the demonstration that a retrieval augmented model can outperform others.

The authors constructed a good case study in that the 19 questions posed represent a good cross-section of typical questions spanning biological, general, and clinical and the use of 8 expert reviewers with a central assessor provides sufficient redundancy to estimate ‘ground truth’ scores.

As is noted by the authors, the study does have limitations – the number of questions assessed is relatively speaking small (N=19); the focus on a sole disease area begs the question of how well these findings would generalize; the better performance may be driven by choices made in the scoring matrix, e.g. relative over-weighting of penalizing hallucinations over incomplete answers.

This is a good point. To address any inherent biases encountered when scoring questions with a single reviewer, we have added an additional two independent reviewer evaluations for each question to assess consistency across scores (Methods, lines 336-341). The manuscript figures have also been updated to account for mean and variance of accuracy, relevance, and readability scores (Figure 2, Supplementary Figure 1), and the increased number of reviewers and scores across all questions (Figure 2, Table 5). We have also mentioned in the Results section (lines 195-197) and in the Discussion (lines 248-255) limitations with the question set and scoring system, which hopefully further acknowledge Reviewer #1’s comments. In the Discussion (lines 220-231) we’ve also pointed out that components of the RAG model are also evolving, and this may affect the quality of answers to questions such as those posed here. Regarding hallucinations, we tried to clarify how we were qualifying a hallucination in the Results (lines 185-190) and Methods (lines 345-351). We also modified our tracking of hallucinations to account for any answers with one or more hallucinations (Table 6).

In addition to the limitations noted by the authors, the paper raises other questions: what is the variance in the expert reviewers’s scoring and how does this relate to the variance in model performance for specific questions? For the retrieval augmented model, how does performance correlate with parameters, e.g. k used in document retrieval or the literature corpus supplied?

This is a good point. With respect to variance in scores, we’ve added additional reviewer scores for accuracy, relevance, and readability for each question (Methods, lines 335-342, modified Figures 2, 3) to mitigate biases in score interpretation of a single reviewer. Regarding the RAG model, we tried to clarify in the Methods (lines 326-333) the distinction between the corpus size and the content within the corpus used to generate an answer. The Discussion (lines 258-269) also now emphasize how the corpus size could affect an answer generated by the RAG model.

The paper would benefit from these additional analyses.

In summary, the authors present a case study of general interest to biotech/pharmaceutical audiences who are keen to reduce to practice LLMs for knowledge base purposes and provide an implementation of retrieval augmented models that could be used widely. The paper is clearly written, the findings substantiated, and the methodology is written such that others practiced in the art could reproduce it.

We appreciate your feedback and hope the modifications and additions we’ve made to the manuscript better address the points you have raised.

I support publication with revision.

## Reviewer #2:

In general, the idea is clear and presents a solution to a common problem in LLMs such as hallucinations. However, the following comments stand out:

Minor comments:

- In the introduction, on line 60, the concept of embeddings is introduced, however the introduction is abrupt and without context. I would suggest introducing the concept of embeddings in introduction, where the concept of retrieval-augmented is introduced so that the reader can have a context of what embeddings are and their use in this specific use case.

This is a good point. In the Introduction (lines 71-83) we have expanded on the concept of embeddings and how they fit into the LLM architecture.

- Figure 2 can be confusing, I would suggest restructuring it in another way (e.g. bar plots) or if necessary move it to supplementary.

Thank you for this feedback. Given other additions to the scoring of LLM answers, we’ve modified the 3 figures in the manuscript. Figure 2 now hopefully more clearly shows the performance and variance of these LLMs in answering the 19 questions posed here. Figure 3 now shows a distribution of scores from each reviewer across the 3-point scoring scale.

- It would be important to know more details about the experiment so that it can be replicated as best as possible. Values such as the temperature of the models, or the value of k used to choose the top k document segments.

Thank you for this feedback. In lines 324-333 of the Methods section, we clarified the k used in the RAG model to select top document segments. For this experiment we settled on a k = 5. Initially we tried k = 1, 5, and 10. The quality of answers did not improve drastically when comparing k =10 vs. k = 5, and in most cases the same sets of papers were being retrieved to craft the answers. We have also included a tab in the supplemental data (excel file) to show RAG answers when varying k.

- Although this is a method that improves the performance of existing language models, it should be mentioned in the discussion that the use of generative language models is still far from being a reality in such clinical practice.

We have now added in the Discussion (lines 284-287) content which emphasizes that additional research and understanding would be required before LLMs are used in routine clinical practice.

Major comments:

- One of the greatest weaknesses of generative language models is the poor capacity for reproducibility and determinism in their responses. For this reason, it is suggested to run the experiments more times and capture the differences in responses. Likewise, a more rigorous statistical analysis of the results is suggested, presenting confidence intervals and statistical tests.

This is a fair point. We have now expanded the evaluations to three reviewers for each question (with the same three metrics per review). With these independent evaluations by multiple reviewers per question we are now also reporting mean and standard error for each scoring category across all 19 questions (Figure 2), as well as a distribution of all scores from all reviewers across 3-point scores (Figure 3). Text describing this was added to the Methods section as well (lines 335-340) .

- Although mentioned in the discussion, the possible biases and limitations in the approach should be expanded. The use of scientific articles from PubMed as a data source may not represent all populations, mainly populations less represented in scientific research, such as populations from low- and middle-income countries (LMICs), and therefore, although the approach reduces some limitations of LLMs such as the retraining needs, we must also be very careful as it could introduce other biases. Likewise, some of the questions must represent this type of problems so that rare cases are also evaluated.

This is a good point. We have added a paragraph in the Discussion (lines 270-280) emphasizing inherent biases in the training data used for both general purpose LLMs as well as RAG models. The well-known biases in LLMs around the issues you point out are important to highlight in this work, and we hope the additional content in the Discussion makes this point clear.
